# Traveling Letter, No. V

**Published:** 1844-10

**Authors:** 


### THE WESTERN JOURNAL.

New Series.—Vol. II.—No. IV.

LOUISVILLE, OCTOBER 1, 1844.

### TRAVELING LETTERS FROM THE SENIOR EDITOR.

NO. V.

Missouri River, August 28, 1844.

*To my Colleagues:*

Gentlemen:—As I hope, to-morrow morning, to pass out of the Missouri river, on the banks or bosom of which I have spent the last five weeks, I avail myself of the landing of our boat, for the purpose of sounding the channel in advance, to begin another epistle intending to finish it when we lie by to-night. It will, no doubt, seem remarkable to you, that a river nearly 2,000 miles long, which in June buried up its highest banks, and swept off all the embellishments, with which the hand of civilization had sought to mitigate its savage aspect, should, in August, require to be sounded, in certain places, before a common steam packet can be allowed to descend upon them. But in fact, this river, which has been called the 'greatest of tributary streams,' is for navigation, inferior not only to our beloved Ohio, but even to the Alabama, the Yazoo, and the Illinos, except in length. This exception, moreover, scarcely extends to the first, which is, perhaps, navigable for 1,000 miles, through a longer period of the year than the Missouri. The truth is, that this boasted child of the Rocky Mountains has long since ceased to excavate its channel, and contents itself with bringing down sand from the tertiary plains through which it flows, and moving it slowly forward from one place to another; thus barring up its own channel, and keeping itself forever superficial; in which respect it may be compared to some of us medical philosophers, who exhaust our energies of mind in shifting from one theory to another, instead of descending into the profounder depths of science. And the parallel does not stop here; for as the river, in the spring-tide of its prosperity, may tempt the voyager far into the wilderness, to wreck him in returning on sand-bars or snags which lurked beneath the swollen waters, so the inflated rhetoric with which some of our systems of medicine have been buoyed up, has too often led the unwary into dangerous quicksands of practice. But our captain has returned from his soundings, and the vibration of our steamer compels me to stop my professional moralizing.

Lord Chatham, or some other great man, it is said, told his son, that it was better to know the character of one living, than of a hundred dead statesmen; and by a sort of analogy, I am ready to say, that one observation written down as soon as made, is worth a multitude, recorded at some after time, should such time indeed ever come. In our own profession, it is peculiarly important, that observations should be written down, while they are yet fresh in the memory—while they still linger in the eye, and ear, and finger ends, as sensations, but it is well known that such is not the 'custom of the country,' and this may all have resulted from ignorance on the part of the graduates of our various medical schools of the *recipe* for steamboat writing which I have just hit upon, and would give as follows: choose the front part of the cabin, near the bar and card tables, and having placed, as I now have, a back-gammon board on your knees, proceed to make 'the necessary passes.' Henceforth they will be able to commence recording, as soon as they are authorized to commence observing; and may, on their returning voyages, form habits that will abide with them for life. The first acts of a graduate are apt to be his precedents through coming years; for there is no era of his life in which his self-complacency is so exalted, as the time which passes between receiving his diploma with its blue ribbon, and receiving crape and gloves, to wear at the funeral of his first patient. I would, then, that every young doctor should begin to make notes as soon as he finds the steamer which is to bear him to the theatre of future action, fairly at the top of her speed. He would thus begin, as he should continue and end. Let it not be said, that he would find nothing on a steamboat voyage worth recording. If he should not, it would be because he did not observe; and this leads me to say, that too many of us are inattentive and inaccurate observers, when, above all other men, we should be most acute; for medicine is a science of observation. But he who records most, will observe best; and the method I am proposing, will contribute to form early habits of observation as well as registration—to use the shortest word which occurs to me. Still further. The young physician who is bent on studying the physiognomy, and physiology, and habits of his fellow voyagers, and indulges a taste for the scenery of nature through which he moves, and records the things which are new, and the reflections they raise in his mind, will feel little taste for the fascinations of the gaming table, by the side of which he may sit, noting down what passes in the world around him; nor will the fumes of glasses, with which black-legs and gentlemen maintain their courage under the agitations and losses of the game, raise in him any inordinate desire. Thus, you see, I have put forth a *formula* for making good as well as great men, and will now give you some accout of a *formula* designed to preserve their lives.

For the last fortnight, I have been in the neighborhood of the celebrated *Dr. Sappington*, of whose 'anti-fever pills' you have heard, but whose book, recently published, you have not perhaps seen. Its title-page is as follows: 'The Theory and Treatment of Fevers, by Dr. John Sappington, of Salina county, Mo. Revised and corrected, by Ferdinand Stith, M.D., of Franklin, Tennessee. Paul may plant and Apollos water, &c. Arrow Rock, Missouri; published by the author, 1844.' I have given this work a perusal, and remark concerning it—1st. That exclusive of preliminary matter, it consists of less than 200 small, open printed, duodecimo pages, and yet is put at the price of *two dollars*—of course it is a work of great value. 2d. That it embodies the certificates of 150 citizens of Missouri and other States, in support of its author's character—which of course must be uncommonly good. 3d. That while its author classes himself with the profession, and modestly tells us of teaching Professor Barton, 30 years ago, how to cure ague and fever with bark and opium, his work is addressed to the people, and abounds in sarcasm and censure on educated physicians, designed to discredit them in the public estimation. 4th. That he has put forth nothing new, not even a new error of any magnitude, but, embodying a few chapters of common truths, with a number of of venial errors, has offered them to the people as an original system, and warned them to confide no longer in the very works from which he has derived the materials for his own. 5th. That he has referred to the ultraism of certain individual physicians of different ages, as if it had been that of the profession. 6th. That like his prototype, Dr. Samuel Thompson, he has not invented or discovered any new medicine, but taken those already known to medical men, and sought to make money by the sale of them as new articles, or old, administered in a new and more effiicacious mode. 7th. That while Dr. Thompson got a patent and made the patentee purchase his book, Dr. Sappington concealed the composition of his pills, and seeks to make the people purchase his work, at four times the current price, for the purpose of learning how to make them. 8th. That their composition is as follows: '*Sulphate of Quinine* 40 grains, *Gum Myrrh* 10 grains, *Liquorice* 30 grains, *Oil of Sassafras* sufficient to give them an aromatic smell. Mix and make 40 pills.' One to be taken every two hours. The book informs us, that it is now ten years, since the sale of the pills commenced; during which a million of boxes have been sold—amounting of course to a million of dollars, and affording, I have been told, a net profit of about three-fourths of that sum! Now the work tells us, truly, that the sulphate of quinine is the only active ingredient in this compound; but the administration of that medicine in grain doses, for the cure of intermittent fever, was universal, in this country, ten years before Dr. Sappington labeled his first box. His merit then consists in filching from the profession one of its standard medicines, and converting it into a secret panacea, for the purpose of making money by its sale to those who prefer to take that of which they know nothing, with printed directions, to that which they know, with directions in writing. As this class make up the majority of the world, the sale, as a nostrum, of a valuable medicine, is no marvel. The materia medica, in fact, contains many other articles, which by selfish physicians, regardless of what is due to their profession and society, might be made to run a similar, if not as successful a career. The efficacy of Dr. Sappington's pills, which, in the general I do not deny, depends on this, that the sulphate of quinine, as it is well known, will seldom fail to arrest the paroxysms of intermittent fever; but this was not a discovery by Dr. Sappington. All that he can claim is, that he has made the discoveries of others instrumental to his own wealth. Moreover, his pills are far from being blameless, for they have often done harm, both positively and negatively. In many instances, they have injured, by being given in states of the system which did not admit of their use, and in others, they have been relied upon, when a larger amount of their active ingredients, with other curative means, were required. The former were inflammatory—the latter malignant. The cases in which they have succeeded were such, in general, as would not have proved fatal, but only protracted, had nothing whatever been done. Even in these, the pills have not accomplished all that was necessary, for intermittent fever, treated with them, as with sulphate of quinine, administered by such physicians as do not follow it with the bark, is liable to relapse. I have spoken of Dr. S. and his pills and book, in connection with autumnal fever only; but must now add, that the last comprehends an account of several other diseases, in which he recommends, of course, a grain of sulphate of quinine every two hours. Towards the close of his work we have a short treatise on the materia medica, and several tables of weights and measures copied out of the Dispensatory—the last of which we may presume to be *the* contribution of his reviser and corrector, Dr. Stith.

Dr. Sappington will doubtless regard this passing steamboat notice of his book and pills, as an assault upon him and them, by one of the 'regular profession,' and seek, *perhaps successfully*, to 'make capital' out of it. Well, be it so. As public journalists, it is proper we should notice things as they rise around us, and doing it according to the facts, we are not responsible for the augmented propensity to encourage empiricism, which the people may display. They, good souls, never fail to take sides with quackery against science, and never will, till they know what science is Mean while, all that she can do, is to assert her rights, and then submit composedly to their violation.

Before leaving the Missouri river I must say something of the *Autumnal Fever*, which prevails not only on its banks, but far and wide, in all directions from them. I can hear of no spot, high or low, wet or dry, wood or prairie, village, town or city so called, that is not invaded. It began in June and has not yet, perhaps, reached its greatest prevalence. To find a single family, some member of which has not had a chill or two, would be a curiosity. I was told, to-day, of a village which we passed, in which every individual, except a small negro boy, had experienced an attack! I am happy to say, however, that but few deaths occur. The disease is generally a mild tertian chill and moderate fever, between the paroxysms of which the patients may often be seen on their feet. Their leucophlegmatic countenances, however, betray both their malady, and their gross imprudence. The fashionable remedy, is the sulphate of quinine, to prepare the system for which, some physicians bleed a great deal, others puke, others purge, and others do nothing,—while all claim for their respective methods a good degree of success; and perhaps, in the main, claim it correctly. What they do, neither countervails the antidote, nor gives it efficacy. But this is a general fact, liable to many exceptions, even in this mild epidemic, and would be liable to many more, if it were severe.

From the great prevalence of intermittents, some physicians with whom I have conversed, predict that the present autumn will not bring forth many cases of remittent fever. The general statement is, that the former never prevailed so universally in this country before; and of course it is ascribed to the drenching rains of May and June, and the unprecedented inundation which followed. Since I came upon the Missouri, there has been but little rain or fog, and the air has seemed to be as dry as common.

I must turn from autumnal fever, to a very different disorder, *Club Foot*, that I may do justice to my own merits. I have to tell you, then, and wish to do it with becoming modesty, that I have lately operated twice for that unseemly deformity.

But I must 'hold up,' and bring my rambles to a close. It is now past 11 at night, and I have fairly 'sat out' the gamblers, at an adjoining table, one of whom, I am sorry to say, is a doctor. They have by no means been very mute, and if you find, that any of the technicalities of the card-table, have been incorporated with those of our profession, I hope you will either strike them out, or supply them with synonyms; and thereby,

Oblige your drowsy, yet very
Obedient servant,
D. D.

